# Editorial and Miscellaneous

**Published:** 1862-09

**Authors:** 


### EDITORIAL AND MISCELLANEOUS.

*Cantharides as a Therapeutical Agent.*—Alex. McBride, M. D., U. S. Vol., in the *Lancet* urges that in cantharides the profession have one of the most potent agents "to re-kindle the warning spark of vitality—an agent which in many cases of disease, at an almost hopeless stage, will rally the scattered and almost dissipated vital forces, concentrate and generalize their action and re-establish that series of atomic changes upon which vital action depends.

He argues that the principal effect of the fly vesicant is really attributable to the absorption and general action of the active principle of the cantharides. He has used it in the depressed stages of typhoid fever with pulmonary congestion, in "sinking" chills, in partial paralysis with constipation, in coma with scanty heavy urine, in pneumonia with quinine, in gangrenous erysipelas, in poisoning by animal fluids, in hysteria accompanied by profuse leucorrhœa, in cholera, etc., etc. The general indication for the use of this potent remedy he italicizes as follows:

"*When in atonic, asthenic or adynamic disease it is a desideratum, from whatever cause, to produce general or local capillary tonicity, the internal use of cantharides will be indicated, and in quantity proportioned to the urgency of the demand.*"

Dr. McBride administers the medicine in much larger doses than those ordinarily directed. One fluid drachm, he says, will generally be sufficient, but in severe cases he would not hesitate to double the amount. The effect will be manifested twenty minutes or less. Smaller doses may be repeated in from one to two hours after. No more should be given than sufficient to accomplish the general object, and its administration should not be trusted to any other judgment than that of the physician himself. He admits that it may do mischief when given too largely, or on improper and false indications; but, like mercury, it will do *something* in any dose. From the theoretical and practical considerations he derives the rule: "*Cantharides may be given in free doses* (*in cases where indicated*) *as long as the urine continues of a darker color than pale amber.*"

Strangury does not occur as long as the urine is copious and heavy. When it does occur he regards it as a good symptom. He has seen no evidence of its supposed aphrodisiac action.

We can not look upon Dr. M.'s article as establishing more than the well known fact that the *cantharis* is a powerful but *unmanageable* stimulant. The large doses recommended we have no doubt may be safely employed so long as the kidneys rapidly eliminate the active principle, and precisely there the difficulty commences—failure to eliminate will of necessity entail the poisonous effect.

It is unnecessary to state, after the remarks made last month in this JOURNAL, that we dissent entirely from the author's suggestion that the external application of the fly is beneficial mainly from the absorption of its active principle and consequent systemic impression. Nevertheless it is true in vastly the larger proportion of cases, if not in all, that when the "blistering point" is reached, internal support and stimulation is ordinarily indicated. We regard the article as valuable as being among the many evidences every day accumulating that the deplorable "antiphlogistic" treatment so often carried to death's door, is subsiding to its appropriate level and material disuse. The atrocious paradox of "pulling down with one hand whilst building up with the other"—deemed so profoundly sagacious by some medical theorists—is receiving rapidly its ultimate quietus.

---

*Alcoholic Stimulants in the Army.*—The Cincinnati Academy of Medicine, reported in the same journal, in a discussion upon the subject of army diseases appear to have pretty generally agreed that the judicious administration of alcoholic stimulants is necessary to the health of soldiers in active service. Otherwise the causes tending to enfeeble digestion become still more energetic in action and influence, and concurrently camp diarrhœas, typhoid fevers and conditions, erysipelas, etc., etc., prevail. This is an eminently correct view of the case, and we are out of all manner of patience at that counterfeit and base "philanthrophy" (!) which has robbed the soldier of this physical necessity. It is all very well for dyspeptic parsons to thunder phillipics from their pulpits and coin adjectives and denunciations in their easy chairs; it is all right enough, we suppose, for Congressmen, torpid with costly Cogniac and Champaigne, to pander to a sickly sentimentality among their radical constituents at home, and thus the soldiers be robbed of one of the prime necessisities for health—but, let it be remembared, a fearful reckoning is being made up.

The moderate spirit ration is not conducive to intemperance, but positively a preventive. Whatever the mental habit of the soldier, the body will claim its rights. The articles of war cannot control the facts of physiology and common sense. The Maine liquor law applied to the army results much as it did when applied elsewhere. The pent up physical want of the soldier explodes in a volcanic excess of drunkenness the moment opportunity offers, and the history of this war, as of all wars, shows that opportunities do now and then occur in spite of the "regulations." The natural result is disease from the excess—just as the starving man endangers his life from unchecked voracity, whereas when moderately fed he eats *tuto et jucunde.* The occurrence of disease from the excess is charged upon the stimulant, and "tee-total" objurgations deafen all ears. It is time that this folly should cease. Practically it is being put to an end by some of the more sagacious commanders in spite of "red tape" and the clamors of easy going theorists, wrathfully reforming the world in their carpeted chambers at the cool North. One way is by putting a little Quinine in a comfortable drink of whiskey as a *prophylactic*. Another way is by sending the soldier to the hospital or his home, where clear-sighted medical men know what is necessary and direct it with discretion. In our view prevention is better than cure.

It is to be expected that some dilapidated temperance lecturer, whose mental hull is laid up in ordinary, covered with barnacles, will launch out once more upon the sea of doleful platitudes when he reads what we have written. It is to be expected thot some physiologic *sardine* will writhe and wriggle, in his oily packing, at the remote suggestion that his slumberous beatitude of hybernating humanitarianism is disturbed. But the plain truth is, "teetotalism" is just as applicable in the army as vegetarianism, and the arguments to sustain the one are precisely those to sustain the other. Some men have lived and do live without meat, and some men have lived and do live without meat, and some men have lived and do live without stimulants. Some have died from gluttony and some from intemperance; but eating and drinking may still be set down as consonant with life and health.

It is unfortunate that some medical men will still give way to this depraved sentimentality, just as they still too often listen to the fears of patients and attendants that nutritious food, the first element of success in the treatment of most, if not all, diseases, is dangerous in fevers. The professional follies and delusions of one age infallibly become the popular opinions of the next, and he is a man of more than ordinary influence and strength of mind who can always, or even often, combat them with success.

It is safe to say that this whole subject of alcoholic stimulants needs re-statement by the scientific men of the profession. The contra-stimulant doctrine is already, with the starvation system, practically consigned to "the tomb of all the Capulets."

*Iron in Chlorosis.*—The remark is attributed to TROUSSEAU, that preparations of iron given in cases of chlorosis with a tendency to tubercular disease, favor and hasten the development of the tubercles. It is therefore observed as important that cases of true chlorosis should be carefully diagnosed from pseudo-chlorosis. This opens a rich vein to the medical satirist; but by no means wishing to assume that character, we call attention to the strikingly apparent fallacy involved in the statement. The inference is that chalybeates are useful in the one, and not in the other—that chalybeates *per se* favor in some cases the development of tubercles. As though in either case there was a chance for antidotes, or for adjuvant poison. Such notions only tend to mislead. The chalybeate stands upon the same footing with any other medicine in such instances, and no Gallic refinement of diagnosis can do away with the stubborn fact that if the medicine improves the appetite, digestion and assimilation, it will be useful, whether the chlorosis be "true" or "pseudo"—if it impairs these, it will favor tuberculization. Tuberculosis is not to be met by antidotes, or prevented by ingenious combinations of drugs. The duty of the practitioner is to secure a bath of healthy blood for the diseased part—to see to it that the forming blood be provided with suitable material out of which to build up and repair tissue, an end to be secured, not by specifics based on strained dilettante distinctions, but by careful and comprehensive view of all influences operating on the patient, and restoration of normal physiological conditions. Is it not about time to quit this constant inquiry for specifics? Let the homœopathists have the full monopoly of the nostrum and panacea business.

---

*Local Treatment of Diphtheria.*—DR. WELLER, of Evanston, informs us that he has relied in several cases of severe diphtheria upon local treatment alone, and with marked success. He has found the best result from a lotion of Hydrarg. Bichlorid, one grain to eight ounces of water applied freely to the affected parts every four hours. Next to this, he ranks a dilute solution of Perchloride of Iron. In some instances, alternation of the two at intervals of two hours seemed particularly efficient. He has promised us details for an early issue of the JOURNAL.

---

*Prophylaxis of Diphtheria.*—M. LOISEAU commends as a prophylactic when epidemics of diphtheria prevail gargles or lotions of aqueous spirituous or ethereal solutions of tannin, the strength varying with the age and susceptibility of the patient. A few drops may be swallowed each time. The addition of Chloroform to the alcoholic solution is occasionally useful. There seems to be a great diversity of practice among physicians, based mainly on the fact that some believe diphtheria to be only a local affection, others that it is a constitutional disorder.

---

*Nitric Acid in Pertussis.*—DR. BARKER, in the N. Y. Academy of Medicine, stated that he did not think Nitric Acid was applicable in the first stage of whooping cough, prefering at that period small doses of Tinct. Rad. Aconiti with rubefacients over the upper part of the chest posteriorly and anteriorly. After subsidence of inflammatory symptoms, free imbibition of nitric acid lemonade—fifty or sixty drops of the dilute acid to a tumblerful of water. He recommended also ℞—Syrup Lactucar, ℥j; Ext. Belladonna, gr. $\frac{1}{8}$; Quiniæ Sulphat, gr. ss. M.—To be taken three or four times a day.

---

*Alcohol in Purpural Hæmorrhage.*—Rapid and prolonged intoxication is recommended in the *Gazette des Hopitaux* as a remedy in cases of profuse hæmorrhage depending on purpura A most efficient combination for this purpose is made by adding to the alcoholic stimulant full doses of Sp. Terebinth.

---

*Chlorinated Soda in Scarlatina.*—A writer in the *London Lancet*, July, 1862, reports several severe cases of anginose scarlatina, with typhoid prostration, successfully treated by internal administration of solution of Chlorinated Soda. From twenty to thirty mincins were given in Camphor mixture every hour. The solution was also used as a gargle. Brandy was also given internally in doses of about four ounces daily in one of the cases reported. The Sulphuric Acid was given as a tonic during convalescence. Probably the local application of the solution and the brandy were the really efficient agents. However, as there is always a great anxiety to "give something" in severe diseases, the Chlorinated Soda, in the doses indicated, may be given with as much confidence as the Chlorate of Potash—even though it will scarcely be claimed as "oxygenating the blood."

---

*Arsenic in Onychia Maligna.*—Mr. Curling, in the London Hospital, treated a bad case of onychia maligna of the great toe by first poulticing and removing the slough, and then applying a lotion composed of equal parts of Liquor Arsenicalis and water. This caused severe smarting and burning pain which lasted two hours. Several hours after, it was again poulticed and a grain of Morphia exhibited. The sore was thus treated on four alternate days, the pain caused being less each subsequent application. Strips of lint soaked in solution of Nit. Argent. were then applied, the edges of the ulcer supported, and at the end of a month the patient was discharged perfectly cured. The case had been one of marked severity for a long time, having required excessive doses of opium to alleviate the intense pain—often as much as one or two ounces of Laudanum daily. The local alterative effect of the arsenical solution was very striking.

---

*Lotion for Infantile Erysipelas.*—M. Loiseau has used in many cases of erysipelas around the vaccine pustule in infants a lotion composed of tannim dissolved in brandy, with the addition of a small quantity of chloroform. The application is repeated at first at intervals of ten minutes, gradually increasing the space to half an hour or more. It promptly relieves the pain and checks the spread of the disease. M. Loiseau thinks it probably applicable to erysipelas arising from other causes.

---

*Asphyxia from Chloroform.*—Dr. Wm. Marcet, argues in the *Med. Times & Gazette*, that asphyxia from Chloroform is owing not only to its anæsthetic properties, but to its producing spasm of the glottis. He therefore urges, when this occurs, tracheotomy as well as continuous artificial respiration.

---

*Chloroform in Otalgia.*—Dr. Pattee, in the *Jour. of Mat. Med.*, recommends, in earache, to put a little loose cotton in the bowl of a common tobacco-pipe, drop on it eight or ten drops of Choloroform, cover this with another pledget of the cotton, then place the stem to the affected ear, and blow steadily into the bowl so as to carry the vapor directly into the ear.

---

*Tannate of Iron and Collodion to prevent Pitting by Small Pox.*—Dr. Thompson, at a late meeting of the N. Y. Co. Med. Soc., remarked that at the Quarantine Hospital they used a mixture of Collodion, Tannin and Iron with good effect in preventing pitting; the object of the mixture being accomplished by excluding the light with the black mark thus formed.

It is probable that the various measures proposed accomplish the object, if ever, by diverse methods, some as cauterants by aborting the pustule, others by relieving inflammation directly, and others indirectly by excluding the air and light.

---

*Pernitrate of Mercury in Solution* is strongly recommended by Dr. Gay, of the Great Northern Hospital, in cases of epithelial cancer, lupus exedens, and indurated chancre. The application causes great pain for an hour or two, but when the slough separates, a healthy granulating surface is left. It seems to quicken the energies of the healthy tissue, so that no sooner is the disease gone than the wound is almost cicatrized, and that without the loss of tissue sustained by excision. The application may be made twice a week until all traces of local disease have disappeared.

---

*The Mitigation and Suppression of Venereal Diseases.*—This subject is one which is attracting the attention of the sanitary associations in most of the large cities of Christendom. Many plans have been suggested to meet the evil, but there are practical difficulties in the way of every one which has yet been brought forward. Meanwhile, the steady spread and increase of venereal diseases has become such as to awaken the most serious alarm, not only of the philanthropist, but of the political economist. The columns of the secular papers are rank and offensive with the obscene lucubrations of advertising knaves. Wherever the careful owner of a corner lot has put up the notice "Commit no Nuisance," the injunction is fearlessly disobeyed, by numberless posters of "Sacred Retreats," "Lock Hospitals," and "Confidential Advisers," warranting speedy cures without mercury. Fortunes are amassed by these vultures, whilst the healths and constitutions of their dupes are left in ruins. Those who have not examined into the subject, have no conception of the frightful waste of property, morals, health and life involved; but a morbid public sentiment still prevents proper action in the premises. Publication of the appropriate methods for mitigating and suppressing this master evil, is out of question. It can be met only by a quiet but relentless despotism—a despotism which can easily be so regulated as only to affect the evil doers—the fountains of this corroding pestilence. Boards of Health and Sanitary Police must assume power in the premises, and the evil can readily be shorn of its enormous proportions.

---

*Penetrating Wounds of the Brain.*—Recent cases have robbed the "crowbar case" of its solitary pre-eminence. Beaumont, of Toronto, reports a penetrating wound of the brain, produced by a broken rocket shaft through the left orbit. The stick was extracted from the part with great difficulty. It passed almost directly backward nearly parallel with the mesian plane, apparently through the sphenoidal fissure, and into the base of the middle and posterior lobes of the left hemisphere of the brain. Its extraction was followed by a profuse rush of blood, a stream almost as large as the extracted shaft; but the bleeding almost ceased in five or ten minutes, iced water being applied over the forehead and upper part of the face. He did not even faint, and in fifteen minutes got up and walked. With the exception of application of iced water, and strict rest, little or nothing was done or ordered. Recovery was complete, with the exception of loss of the eye, paralysis of the parts supplied by the infra orbital branch of the superior maxillary nerve, and some imperfection of memory of matters which occurred subsequent to the injury.

In an instance recorded by Dr. A. H. Stephens, of the 6th Ohio Volunteers, in the Cincinnati *Lancet*, a minie ball proved almost as harmless as a homœopathic pill. We quote from the Doctor's account:

"One man was carried in on Monday. I could learn nothing of his case save that he had been found on the field insensible. He was perfectly comatose. On examination, I found an opening through the scalp and cranium, into which I introduced my finger full length. Finding the cavity filled with fractured bone, coagula, etc., I enlarged the opening of scalp to the extent of two or three inches, and removed a portion of the lower border of parietal and a larger part of mastoid process of temporal bone, then introduced the forceps and removed an ounce Minie, which had buried itself about two inches within the brain; but in removing the ball about three ounces of brain matter escaped. I replaced the integuments and dressed the wound as well as the case would admit, and placed the patient in his bunk to die; but in twenty-four hours his coma had quite left him, and he was rationally and loudly calling for water and indulging in many soldierly expletives to all who did not instantly attend to his wants! At the expiration of seven days, when he was placed on board of a boat to be transferred to a hospital, he had every appearance of a perfect recovery without the slightest relic of paralysis, and he informed me confidentially, that in four or five weeks he would return to 'crack the d—d butternut that hurt his head.'"

This occurred at the Shiloh battle. Verily, we shall begin to believe that brains are of little use in war-time.

*En passant*, we have information of a case where a surgeon poked a probe around in the brain, in search of a *Minie*, with a *sang-froid* that astonished the bystanders, but this man *died*. The surgeon recovered.

---

*Amputations in Military Hospitals.*—Dr. Sanford B. Hunt writes the Buffalo *Med. Journal* that operations on the field have proved more successful than secondary operations, adding but little to the general shock, even when high up on the thigh. Yet, he says, there are a vast number of exceptions, which should be borne in mind by the ambitious field operator. Thus cases of bullet wounds of the upper third of the thigh where the missel is buried in the femur without absolutely fracturing it. So also in wounds of the joints, especially of the elbow, which somehow endures disaster better than any other. Referring to the exploits in hospital of certain "distinguished operators," he eulogizes Brigade Surgeon John W. Hunt,.of Western New York, (a namesake, but not a relative, of his,) who, on taking charge of the Mill Creek hospital, carefully locked up the surgical instruments and relied on a pair of scissors and a forceps. He certainly killed nobody, and the reporter thinks largely decreased the mortality, besides saving many useful limbs. He states that the conservative surgeons at Fortress Monroe trusted very little to resections, and did not hesitate to condemn their frequent employment. The proportion of operations to patients in the hospitals he says scarcely averages one per cent. "It remains to treat the majority *pro re nata*, to apply cerate to the kindly wounds, and water dressings to those inflamed, to watch carefully their cleanliness, to support with wines and tonics under exhausting discharges, to temper irritability with opium, and to secure for them as good a diet and as pure an air as circumstances will permit."——Which convinces us that Dr. S. B. H., whilom editor of the Buff. *Med. Jour.*, has progressed since he wielded a critic's pen some eight or nine years ago. The war is having some good influences at least on the profession. The importance of nutritious diet and judicious use of opiates and stimulants is now pretty generally recognized among surgeons. Free ventilation and absolute cleanliness are also gaining in favor among them, although still almost wholly ignored by barrack builders, (witness the cattle sheds called barracks at Camp Douglas!); but, alas, physicians too often still imitate the boiling oil of ante-Pare times, or the healing salves, knives and saws of later times. Whereas the truth remains that the internal wounds produced by disease, about equally necessitate the same things. But patience!—the world moves—*Speculo ad alteram.*

---

*Excision of Joints.*—In a paper read before the Royal Med. and Chirurg. Society, by Dr. G. M. Humphrey, on the influence of Paralysis, Dis. of Joints, &c., on the growth of the bones we find the following conclusion, among others, of practical interest:

"*Excision of the knee*, if the epiphysial lines are removed, is followed by marked arrest of growth in all parts of the limb. If the epiphysial lines are spared, the growth, in most instances, keeps pace with that of the opposite limb." Preceding diseases, or invasion of the epiphysial lines by the suppurating process may modify this statement. Nevertheless the fact is so constant that every effort should be made to spare these lines, so that the limb, especially in young and growing subjects, may reach its maximum of perfection. In some instances the possibility or impossibility of choice in this respect may determine the selection between exsection and amputation.

During the discussion of this paper before the Society, Mr. Barwell and others adduced cases where local abscess had destroyed the epiphysial lines, resulting in permanent loss of elongating growth in the bone. Paralysis, anchylosis, &c., probably diminish the growth of the bone and soft parts by the loss of exercise involved.

---

*Iliac Aneurism.*—Professor Syme, of Edinburgh, recently remedied a case of iliac aneurism by opening the sac and tying the common iliac and both the external and internal iliacs. A screw clamp of peculiar construction, provided by Prof. Lister, of Glasgow, was employed to effect compression of the abdominal aorta, and when this was complete the sac was opened through all the textures concerned, by a free incision, and about six pounds of blood and clots scooped out. The ligatures separated on the nineteenth day, everything having progressed in the most favorable manner. It may be remarked that the ligatures were applied close to the sac—Prof. Syme observing that it was no more likely that the artery was diseased near to than remote from the rupture. This bold and decisive assertion, justified in its successful result, was strongly urged by Prof. S. as negativing Hunter's supposed universal rule—as for example, in axillary aneurism, where the vessel is easily reached at the point of rupture, and with great difficulty, as well as danger, tied above the clavicle. The modification of practice in such cases is altogether analogous to Mr. Guthrie's ligation of arteries at the part wounded rather than at a distance on the proximal course.

---

"*Bock Agen.*"—"Monsieur Tonson has come again." The Brigadier still claims the benefit of our columns for advertising purposes. We believe our correspondent must have been mistaken when he spoke of him as being of the German persuasion. At all events, he has lived in Yankeedom long enough to be up to the latest Yankee advertising dodges. Notoriety, the Brigadier evidently believes, is the next step to fame. Once to have people read his name in print, and then, by a *ruse de guerre*, make them believe the animal is a true lion and not the other quadruped, or "Bottom the weaver." The actual cautery applied to the Brigadier's back first, sent him braying and prancing up and down the columns of the Ex-Governor's paper as the victim of his "nationality."

That dodge was found not to work, although he scattered his melancholy lucubrations into the possession of every surgeon, suspected of being an "adopted citizen," throughout the grand army. The surgeons of adopted citizenship saw through the leonine skin, and recognized the unmusical bray.

Still writhing under the unappeased smart, a contemporary has hitched him to the ambulance of their college and tries to make him do duty in taking not only himself, but them, out of the field—trusting that his struggles might prove more successful than those of their recently discarded patron saint. It won't do!

In the multiplicity of demands upon our correspondent's time he finds no present opportunity, even did he deem it necessary, to reply to the elaborate tissue of misrepresentations which the Brigadier and his associates have so wearily woven. Too many cooks have spoiled that broth—there is no coherency or compatibility to its parts.

The Brigadier says at the outset that he did not have charge of that boat; but, as he afterwards states that he had, and and gives details to show that he had, the Brigadier simply contradicts the Brigadier.

The Brigadier says that the Chicago *Tribune* failed to return either his communication or his postage stamps. We commiserate the Brigadier, and candidly admit that the *Tribune* folks ought to return his stamps.

The Brigadier says he scarified the eyelid to relieve the œdema and intense pain with which the patient was continuously screaming, and not to evacuate pus. Our correspondent is prepared to prove by affidavits if required, that the patient was not screaming, had not screamed and did not scream until the Brigadier incised and *squeezed* the part; that the Brigadier asked our correspondent if he did not think there was pus in the part, and without waiting an answer enlarged the wound "near the left eyebrow"—not in a depending part, but above the accumulation, whatever it was. In common with all good surgeons, our correspondent believes that incising an eyelid to relieve œdema is bad surgery always.

The Brigadier writes that our correspondent had some degree of responsibility about the application of some splints to a German soldier with a femur fractured by a gun-shot. The only thing our correspondent had to do with the case was in assisting Dr. Gillette to apply some splints the latter was agent for. He had first applied some short splints, there being none other to be had. All the rest of the Brigadier's statements upon this point are simply false, and his insinuations dastardly.

It appears, also, that the Brigadier never heard of trephining the spine, and ridicules the idea of searching for a ball which might have depressed one of the vetebral laminæ. In the case alluded to there was paralysis of the sphincter ani, bladder and lower extremities. In accordance with the most importunate entreaties of the patient, he was anæsthetized and search made for the ball with the bare chance that it might be reached and the patient be saved. Without the operation, death was inevitable—with it, there was a remote hope. There was no bloody cutting, no squeezing, no screaming—but a painless sleep during which there was an attempt to give a patient his last and only chance. The result was unsuccessful, but the attempt none but a donkey, (or a Brigadier,) with his hide still smoking from the branding iron, dare say or insinuate shortened life an hour.

The notoriety-seeking Brigadier says that the Medical Director, understanding his lamentable physical condition, sent "a very respectable medical officer" to assist him. This very respectable medical officer was Dr. Stahl, of the 10th Illinois. This identical gentleman told our correspondent that the Medical Director sent him because the Brigadier was incompetent —what further he said or did might be written, but the Brigadier might as well drop it here.

Our correspondent was informed in so many words by Dr. Isham, of the Sanitary Commission, that he was to take charge of the boat, and accordingly went to work systematizing matters. He was brought up with a round turn by the Brigadier asking the Medical Director who bad charge of the boat. "You," said the Director, whereupon our correspondent subsided. Up to that time our correspondent had not seen the Director, so that if any unfavorable influence had been exerted on him, it was not by our correspondent.

The getting off of the boat was *verbatim* as described by our correspondent. The Brigadier, in denying it, shows that either his memory or his moral faculties have been impaired by his "*wounds.*"

The Brigadier winds up his advertising circular by triumphantly asking our correspondent why he does not join the army? We have asked the same question, and received this reply:— "Because I am afraid I should get to be a Brigadier—should get on my horse some nice day and ride leisurely on to the battle field, a shell should explode, scare my horse, he fall down on me and tear my cap, and hold me until I was "out-worn to death nearly," should then have to be ambulanced to a convenient boat to get home; "go lame" when any of the medical or other officers were about and go straight when they were not; come home and stay several months on pay while I was doing nothing, instead of resigning for the good of the service when I found myself worthless in it; should devote my leisure to writing scurrilous articles to the secular and medical press, when I ought to be posting upon the army regulations, and perhaps moderately on ophthalmic surgery."

Candidly we think our correspondent more than half right. We have devoted more space to this Brigadier than we intended; if he wishes further notice he must get it elsewhere, even though he seizes an initial as the excuse for attacking the position of a Professor in Rush Medical College. In our humble opinion a teacher in a respectable medical college at the present time occupies a position which in responsibility and importance is second to no other. The point does not need argument; military schools are trivial in importance beside the medical colleges. As a teacher of Anatomy, the tongue of the rankest calumniator dare not whisper a word in detraction of the splendid abilities of the gentleman whom the Brigadier singles out as the object of his venomous spleen. So far as any question of veracity between the two is concerned, we scorn to insult our correspondent by making the slightest observation. So far as the nationality is concerned, we have only to say that our correspondent, whatever his ancestry, knows in the United States but one country and one people, and if he takes the money of the Government it will only be as the scant recompense of arduous service, and not as a douceur and pension for trivial or imaginary hurts.

But, what has Rush Medical College to do with the controversy between the Brigadier and our correspondent? The petty malice which could lug in the low-flung denials and vituperation of the cauterized Brigadier to point an attack on a position in the College, is of a piece with that same brilliant strategy which has led its originators, when defeated before the medical public, to seek to bolster up their tottering fortunes by secret political intrigues and kitchen-cabinet chicane. The same pitiful back-stairs influence in the purlieus of Springfield which has resulted in the profession being un-represented wherever its power should have been felt, breathes all along the lines of the retired Brigadier, nursing a wound which the hot iron of our correspondent did unexpectedly make in his pachydermatous integument.

Honorable medical men should scorn such petty artifices and despicable devices. We notify the Brigadier, and his backers, that the attempt to throw dirt at the Medical College by his pawings and kickings, will prove as futile as his previous attempt to cover his blundering inefficiency under his nationality.

---

*Surgical Instruments.*—We are frequently consulted by subscribers in different sections of the country, with reference to facilities in Chicago for procuring surgical instruments of various sorts. Very many wish to procure particular instruments, or to make up cases differing in some respects from the usual ones, or again to have old instruments or cases repaired, replaced or refitted, or new instruments for particular uses. To save ourselves time, and also to communicate what we believe will prove of value to our readers, we here put down that Tolle & Degenhardt, of 130 Clark St., whose advertisement appears regularly in this journal, are a firm who can be fully relied upon in every respect in the premises. They employ none but the most thoroughly competent workmen, and every particle of material is most carefully selected. They use only the finest German steel of Staub, and every blade, or other manufactured article, is fully warranted. We apprehend from our experience in their wares, which has been considerable, that they have little or none returned upon their hands. It is totally unnecessary for surgeons in the North-West to send to the far East for instruments or apparatus, whilst such ingenious and reliable artisans are among us. We heartily commend them and their works to correspondents and readers. With very little noise or attempt at display, they have already built up a business which is not only creditable to themselves, but to our city.

---

*Conservative Surgery.*—A correspondent is preparing the details of a case where the wheel of a railroad car loaded with wheat, passed over the ankle of a boy of some dozen years of age, and yet, not only the foot, but the ankle-joint is saved and useful. The accident occurred about a year since in this city.

---

*Rush Medical College.*—It is with great gratification that we are enabled to announce that, in the possible contingency of the absence of Prof. Blaney the ensuing session, Prof. E. S. Carr, of the University of Wisconsin, has consented to give the course on Chemistry. The national reputation which Prof. Carr has achieved by his lectures on this branch, whilst connected with the Medical Colleges of Castleton, Albany and Philadelphia, renders it unnecessary to introduce him to the friends of the College. For profound learning, success as a teacher, brilliancy and eloquence as a lecturer, he is unsurpassed—we had almost written unrivalled. The great demand for qualified medical men now experienced throughout the country, renders the prospects of the College at present peculiarly flattering. We congratulate the coming class that the hiatus caused by the absence of Prof. Blaney is to be so happily filled.

Owing to the absence of Dr. E. Powell, as Surgeon of the first Board of Trade Regiment, it had become necessary again to supply the place of Demonstrator of Anatomy. Students who were present the last session will learn with great satisfaction that I. P. Lynn, M. D., who then filled the position with such marked ability and success, has consented to act in the same capacity the ensuing session. Dr. Lynn demonstrated then his peculiar adaptation to the position, and by his thorough acquaintance with the subject, his energy, zeal and personal qualities contributed largely to the general success of the course. As a general practitioner—as surgeon of a regiment—as Demonstrator in the College, indeed in every responsible position where he has been placed, Dr. L. has invariably gained distinction for himself and reflected honor upon Rush Med. Coll., of which he is an alumnus. We therefore announce the renewed connection with peculiar satisfaction.

---

*Dr. Fisher's Case of Ovariotomy.*—A little derangement of our calculations as to the space occupied by matter already gone to press, prevents our noticing in the present issue Dr. Fisher's remarkably successful case of ovariotomy. We shall take occasion to comment upon it the next month. The writer having previously diagnosed the case, and strongly advised the operation, has naturally felt much interest as to the result. It is due to Dr. F. to say that the extraordinary immunity from unpleasant or disastrous sequelæ was largely due to his most constant attention and supervision subsequent to the operation. This is the point of almost supreme importance.

---

*Conservative Medicine.*—The attention of all thinking, progressive medical men is requested to the exceedingly able essay, by Prof. Flint, which is commenced in this number of the Journal. Sustaining, as it does, views which for years have been incorporated in our teachings, written and didactic, we hail it as eminently fit to be read and pondered.

